# Supplementary material for: From symptom to cancer diagnosis: Perspectives of patients and family members in Alberta, Canada
Source: PLoS One. 2020 Sep 24;15(9):e0239374. doi: 10.1371/journal.pone.0239374 (PMC7514000; doi:10.1371/journal.pone.0239374)
Supplement: S1 Appendix — (DOCX) [file pone.0239374.s001.docx]

**Appendix. Semi-structured interview protocol (patients)**

1. *Patient identification of symptoms (‘appraisal interval’)*

I would appreciate if we can go through your cancer story, right from the very beginning: At some point you felt something was not quite right, can you tell me about what happened before you went to the doctor for your first visit?

- What did you notice?
- How did you feel at that point?
- How is it that you decided to go see your doctor?
- Did you do anything to cope with symptoms or discuss symptoms with anyone before going to the doctor?
- For how long did you know about the symptoms before deciding to go to the doctor?
- In your opinion, do you feel there was anything that influenced the time elapsed between the moment you felt a bit off and when you decided to go see the doctor?

1. *First consultation(s) with doctor (‘help-seeking interval’)*

What happened between the time you had already decided you would go to see a doctor and the time you went to see a doctor? What are the different steps you followed?

- How did you feel about going to the doctor?
- How was the first visit? What happened? How did you feel?
- Did the doctor or somebody else give you an understanding of the next steps of the diagnostic process (including a rough timeframe) at this visit?
- In your opinion, do you feel there was anything that prolonged the time between when you decided to go to the doctor and you saw the doctor in your first appointment?
- For how long did it take you to get an appointment since you decided to go to the doctor? How long?

1. *Investigation, referral(s) and appointment(s) (‘diagnostic interval’)*

Now we’ve discussed your symptoms, and your initial visit(s) to the doctor, I’d like to know what happened after that and until you had a diagnosis. Could you tell me all the steps you went through (e.g., tests, follow-up, referral to a specialist)?

*Overall experience*

Overall, from the time you first noticed something was not quite right, what do you think would have helped you have a better experience?

Something important thought all these stages (from noticing to visiting the family doctor, from that visit to getting tests, and from there to getting the diagnosis) is emotional support. Do you think the emotional support you received during these different stages was enough? Would you have benefited from more emotional support?
