# Supplementary material for: From symptom to cancer diagnosis: Perspectives of patients and family members in Alberta, Canada
Source: PLoS One. 2020 Sep 24;15(9):e0239374. doi: 10.1371/journal.pone.0239374 (PMC7514000; doi:10.1371/journal.pone.0239374)
Supplement: S2 Appendix — (DOCX) [file pone.0239374.s002.docx]

**Appendix. Semi-structured interview protocol (family members)**

1. *Patient identification of symptoms (‘appraisal interval’)*

I would appreciate if we can go through your [patient relative]’s cancer story, right from the very beginning: At some point she/he felt something was not quite right, can you tell me about what happened before she/he went to the doctor for her/his first visit?

- What did she/he (/you) notice?
- How did she/he (and you) feel at that point?
- How is it that she/he decided to go see your doctor?
- Did she/he do anything to cope with symptoms or discuss symptoms with anyone before going to the doctor?
- For how long did she/he know about the symptoms before deciding to go to the doctor? How long?
- In your opinion, do you feel there was anything that influenced the time elapsed between the moment she/he felt a bit off and when she/he decided to go see the doctor?

1. *First consultation(s) with the doctor (‘help-seeking interval’)*

What happened between the time your [patient relative] had already decided she/he would go to see a doctor and the time she/he went to see a doctor? What are the different steps she/he followed?

- How did she/he (and you) feel about going to the doctor?
- How was the first visit? What happened? How did she/he (and you) feel?
- Did the doctor or somebody else give her/him (and you) an understanding of the next steps of the diagnostic process (including a rough timeframe) at this visit?
- In your opinion, do you feel there was anything that prolonged the time between when you decided to go to the doctor and you saw the doctor in your first appointment?
- For how long did it take her/him to get an appointment since you decided to go to the doctor? How long?

1. *Investigation, referral(s) and appointment(s) (‘diagnostic interval’)*

Now we’ve discussed symptoms and the initial visit(s) to the doctor, I’d like to know what happened after that and until your [patient relative] had a diagnosis. Could you tell me all the steps she/he went through (e.g., tests, follow-up, referral to a specialist)?

- How did she/he (and you) feel about going through these different steps?
- Did she/he or you discuss the different steps with anybody? With whom and how was that conversation?
- Did she/he/you feel informed or understanding why you had to go through all these steps?
- Did she/he/you feel you were part of the decisions?
- Did she/he/you feel supported during the process? By whom? In what ways?
- For many people (patients and family members) the interval between having tests done and awaiting the results or diagnosis is a quite most stressful and emotional time. Was that also the case for you and? What do you think were the factors that mostly influenced your and your [patient relative]’s stress levels?
- In your opinion, do you feel there was anything that prolonged her/him finding out about the diagnostic?
- For how long did it take for her/him to get a diagnosis since you first went to the doctor until you were provided with a diagnostic?

*Overall experience*

Overall, from the time your [patient relative] wife/husband first noticed something was not quite right, what do you think would have helped you have a better experience (for her/him, and for you)?

Something important thought all these stages (from noticing to visiting the family doctor, from that visit to getting tests, and from there to getting the diagnosis) is emotional support. Do you think the emotional support your [patient relative] and yourself received during these different stages was enough? Would you have benefited from more emotional support?
