# Supplementary material for: From symptom to cancer diagnosis: Perspectives of patients and family members in Alberta, Canada
Source: PLoS One. 2020 Sep 24;15(9):e0239374. doi: 10.1371/journal.pone.0239374 (PMC7514000; doi:10.1371/journal.pone.0239374)
Supplement: S3 Appendix — (DOCX) [file pone.0239374.s003.docx]

**Appendix. Coding example**

This example illustrates how specific text segments in one of the interview transcripts were examined for meaning, assigned to emerging codes, and how these codes were described and linked to other codes.

This study participant was a breast cancer patient. When explaining her experience between identifying symptoms and visiting her family doctor, she said:

“I believe that being aware of breast cancer and what it can do pushed me more into working harder for it. That experience that my brother-in-law went through was horrible. So seeing that, I think maybe pushed me more to think ‘you have to push yourself into getting your health care’”.

The first sentence was considered to contain two different meaning units and therefore was assigned two different codes. The first segment “*being aware of breast cancer*” was assigned to the code “**cancer awareness**”. The second segment “*what it can do*” was assigned to the code “**system knowledge**”. The second and third sentences together contain another meaning unit. “*That experience that my brother-in-law went through was horrible. So seeing that, I think maybe pushed me more to think ‘you have to push yourself into getting your health care’*” was assigned to the code “**second-hand experience**”. Finally, the last part of the third sentence was considered to contain another meaning unit. “*You have to push yourself into getting your health care*” was assigned to the code “**self-advocacy**”.

When describing the meaning of the codes, the code “**self-advocacy**” was described as the ability to ask and obtain for what you need. The codes “**cancer awareness**”, “**system knowledge**” and “**second-hand experience**” were defined as elements relevant in patients/families playing an active role in their care, and seeking medical attention. The codes “**cancer awareness**”, “**system knowledge**” and “**second-hand experience**” were placed at the same hierarchical level, below the higher-level code “**self-advocacy**” and an additional higher-level code –not present in the text used for this example- called “**seeking help**”, and related to them by causality. At the same hierarchical level as “**cancer awareness**”, “**system knowledge**” and “**second-hand experience**” there were other codes in a causal relationship with “**seeking help**” including for example “**seriousness of symptoms**”, “**fear of death**” and “**family pressure**”.
