# Supplementary figures and images for: From symptom to cancer diagnosis: Perspectives of patients and family members in Alberta, Canada
Source: PLoS One. 2020 Sep 24;15(9):e0239374. doi: 10.1371/journal.pone.0239374 (PMC7514000; doi:10.1371/journal.pone.0239374)

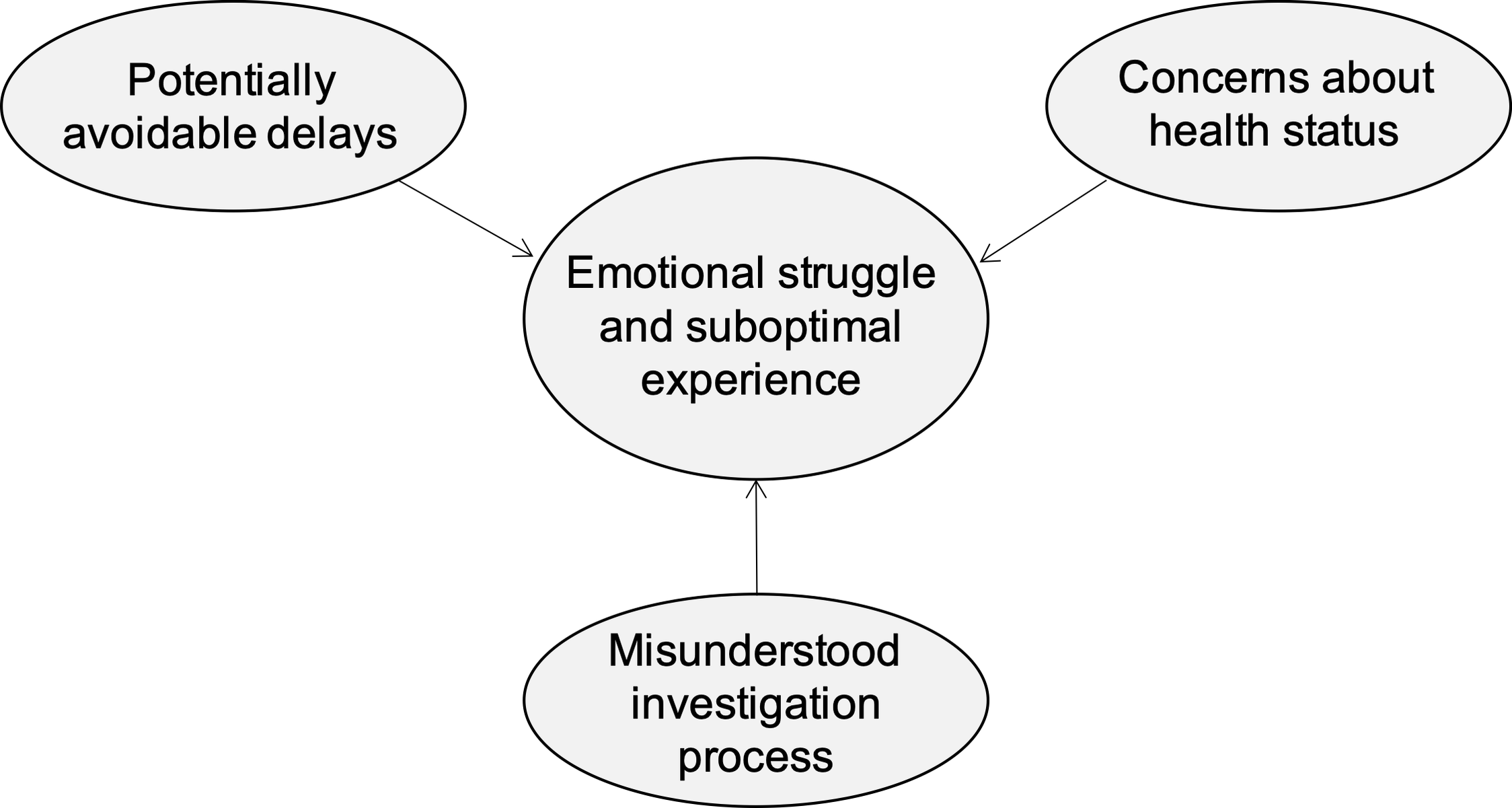

Supplement: S1 Fig — (TIF) [file pone.0239374.s004.tif]
