# Supplementary material for: From symptom to cancer diagnosis: Perspectives of patients and family members in Alberta, Canada
Source: PLoS One. 2020 Sep 24;15(9):e0239374. doi: 10.1371/journal.pone.0239374 (PMC7514000; doi:10.1371/journal.pone.0239374)
Supplement: Attachment — Submitted filename: PONE-D-20-12776_reviewer (1)_Rev.pdf [file pone.0239374.s005.pdf]

PLOS ONE

From symptom to cancer diagnosis: Perspectives of patients and family members in Alberta, Canada

--Manuscript Draft--

**Manuscript Number:**	PONE-D-20-12776
**Article Type:**	Research Article
**Full Title:**	From symptom to cancer diagnosis: Perspectives of patients and family members in Alberta, Canada
**Short Title:**	Perspectives about the cancer diagnosis period
**Corresponding Author:**	Anna Pujadas Botey<br>Alberta Health Services<br>Calgary, Alberta CANADA
**Keywords:**	oncology; cancer care; diagnosis period; self-advocacy; supportive care needs; qualitative research
**Abstract:**	BACKGROUND: Significant intervals from the identification of suspicious symptoms to a definitive diagnosis of cancer are common. Streamlining pathways to diagnosis may increase survival, quality of life post-treatment, and patient experience. Discussions of pathways to diagnosis from the perspective of patients and family members are crucial to advancing cancer diagnosis.<br>AIM: To examine the perspectives of a group of patients with cancer and family members in Alberta, Canada, on factors associated with timelines to diagnosis and overall experience.<br>METHODS: A qualitative approach was used. In-depth, semi-structured interviews with patients with cancer (n=18) and patient relatives (n=5) were conducted and subjected to a thematic analysis.<br>FINDINGS: Participants struggled emotionally. Relevant to their experience were: potentially avoidable delays, concerns about health status, and misunderstood investigation process. Participants emphasized the importance of their active involvement in the care process, and had unmet supportive care needs.<br>CONCLUSION: Psychosocial supports available to potential cancer patients and their families are minimal and crucial for improved experiences before diagnosis. Access to other patients' lived experiences with the diagnostic process and with cancer, and an enhanced supportive role of family doctors might improve outcomes for patients and families.
**Order of Authors:**	Anna Pujadas Botey
	Paula J. Robson
	Adam M. Hardwicke-Brown
	Dorothy M. Rodehutskors
	Barbara M. O'Neill
	Douglas A. Stewart
**Additional Information:**	
**Question**	**Response**
**Financial Disclosure**<br><br>Enter a financial disclosure statement that describes the sources of funding for the work included in this submission. Review the submission guidelines for detailed requirements. View published research articles from *PLOS ONE* for specific	The authors received no specific funding for this work.

Powered by Editorial Manager® and ProduXion Manager® from Aries Systems Corporation

examples.<br><br>This statement is required for submission and **will appear in the published article** if the submission is accepted. Please make sure it is accurate.<br><br>**Unfunded studies**<br>Enter: *The author(s) received no specific funding for this work.*<br><br>**Funded studies**<br>Enter a statement with the following details:<br>• Initials of the authors who received each award<br>• Grant numbers awarded to each author<br>• The full name of each funder<br>• URL of each funder website<br>• Did the sponsors or funders play any role in the study design, data collection and analysis, decision to publish, or preparation of the manuscript?<br>• **NO** - Include this sentence at the end of your statement: *The funders had no role in study design, data collection and analysis, decision to publish, or preparation of the manuscript.*<br>• **YES** - Specify the role(s) played.<br><br>* typeset	
**Competing Interests**<br><br>Use the instructions below to enter a competing interest statement for this submission. On behalf of all authors, disclose any competing interests that could be perceived to bias this work—acknowledging all financial support and any other relevant financial or non-financial competing interests.<br><br>This statement **will appear in the published article** if the submission is accepted. Please make sure it is accurate. View published research articles from *PLOS ONE* for specific examples.	The authors have declared that no competing interests exist.

**NO authors have competing interests**<br><br>Enter: *The authors have declared that no competing interests exist.*<br><br>**Authors with competing interests**<br><br>Enter competing interest details beginning with this statement:<br><br>*I have read the journal's policy and the authors of this manuscript have the following competing interests: [insert competing interests here]*<br><br>* typeset	
**Ethics Statement**<br><br>Enter an ethics statement for this submission. This statement is required if the study involved:<br><br>• Human participants<br>• Human specimens or tissue<br>• Vertebrate animals or cephalopods<br>• Vertebrate embryos or tissues<br>• Field research<br><br>Write "N/A" if the submission does not require an ethics statement.<br><br>General guidance is provided below. Consult the submission guidelines for detailed instructions. **Make sure that all information entered here is included in the Methods section of the manuscript.**	The study was conducted with written ethics approval granted by the Health Research Ethics Board of Alberta –Cancer Committee (Study ID: HREBA.CC-18-0210).

Powered by Editorial Manager® and ProduXion Manager® from Aries Systems Corporation

**Format for specific study types**<br><br>**Human Subject Research (involving human participants and/or tissue)**<br>• Give the name of the institutional review board or ethics committee that approved the study<br>• Include the approval number and/or a statement indicating approval of this research<br>• Indicate the form of consent obtained (written/oral) or the reason that consent was not obtained (e.g. the data were analyzed anonymously)<br><br>**Animal Research (involving vertebrate animals, embryos or tissues)**<br>• Provide the name of the Institutional Animal Care and Use Committee (IACUC) or other relevant ethics board that reviewed the study protocol, and indicate whether they approved this research or granted a formal waiver of ethical approval<br>• Include an approval number if one was obtained<br>• If the study involved *non-human primates*, add *additional details* about animal welfare and steps taken to ameliorate suffering<br>• If anesthesia, euthanasia, or any kind of animal sacrifice is part of the study, include briefly which substances and/or methods were applied<br><br>**Field Research**<br><br>Include the following details if this study involves the collection of plant, animal, or other materials from a natural setting:<br>• Field permit number<br>• Name of the institution or relevant body that granted permission	
**Data Availability**<br><br>Authors are required to make all data underlying the findings described fully available, without restriction, and from the time of publication. PLOS allows rare exceptions to address legal and ethical concerns. See the PLOS Data Policy and FAQ for detailed information.	No - some restrictions will apply

Powered by Editorial Manager® and ProduXion Manager® from Aries Systems Corporation

A Data Availability Statement describing where the data can be found is required at submission. Your answers to this question constitute the Data Availability Statement and **will be published in the article**, if accepted.<br><br>**Important:** Stating 'data available on request from the author' is not sufficient. If your data are only available upon request, select 'No' for the first question and explain your exceptional situation in the text box.<br><br>Do the authors confirm that all data underlying the findings described in their manuscript are fully available without restriction?	
**Describe where the data may be found in full sentences. If you are copying our sample text, replace any instances of XXX with the appropriate details.**<br><br>• If the data are **held or will be held in a public repository**, include URLs, accession numbers or DOIs. If this information will only be available after acceptance, indicate this by ticking the box below. For example: *All XXX files are available from the XXX database (accession number(s) XXX, XXX.).*<br>• If the data are all contained **within the manuscript and/or Supporting Information files**, enter the following: *All relevant data are within the manuscript and its Supporting Information files.*<br>• If neither of these applies but you are able to provide **details of access elsewhere**, with or without limitations, please do so. For example:<br><br>*Data cannot be shared publicly because of [XXX]. Data are available from the XXX Institutional Data Access / Ethics Committee (contact via XXX) for researchers who meet the criteria for access to confidential data.*<br><br>*The data underlying the results presented in the study are available from (include the name of the third party*	All relevant data are within the manuscript. Further data excerpts are available on request from the corresponding author.

and contact information or URL).<br>• This text is appropriate if the data are owned by a third party and authors do not have permission to share the data.<br><br>* typeset	
Additional data availability information:	

Powered by Editorial Manager® and ProduXion Manager® from Aries Systems Corporation

Manuscript Click here to download Manuscript Manuscript.docx

From symptom to cancer diagnosis: Perspectives of patients and family members in Alberta, Canada

Perspectives about the cancer diagnosis period

Anna Pujadas Botey[1, 2 *], Paula J. Robson[2, 3], Adam M. Hardwicke-Brown[3], Dorothy M. Rodehutskors[4], Barbara M. O'Neill[3], Douglas A. Stewart[1, 5]

[1] Cancer Strategic Clinical Network, Alberta Health Services. Calgary, Alberta, Canada

[2] School of Public Health, University of Alberta. Edmonton, Alberta, Canada

[3] Cancer Strategic Clinical Network, Alberta Health Services. Edmonton, Alberta, Canada

[4] CancerControl Alberta, Alberta Health Services, Calgary, Alberta, Canada

[5] Departments of Oncology and Medicine, University of Calgary, Calgary, Alberta, Canada

* Corresponding author:

E-mail: Anna.PujadasBotey@ahs.ca (APB)

Abstract

BACKGROUND: Significant intervals from the identification of suspicious symptoms to a definitive diagnosis of cancer are common. Streamlining pathways to diagnosis may increase survival, quality of life post-treatment, and patient experience. Discussions of pathways to diagnosis from the perspective of patients and family members are crucial to advancing cancer diagnosis.

AIM: To examine the perspectives of a group of patients with cancer and family members in Alberta, Canada, on factors associated with timelines to diagnosis and overall experience.

METHODS: A qualitative approach was used. In-depth, semi-structured interviews with patients with cancer (n=18) and patient relatives (n=5) were conducted and subjected to a thematic analysis.

FINDINGS: Participants struggled emotionally. Relevant to their experience were: potentially avoidable delays, concerns about health status, and misunderstood investigation process. Participants emphasized the importance of their active involvement in the care process, and had unmet supportive care needs.

CONCLUSION: Psychosocial supports available to potential cancer patients and their families are minimal and crucial for improved experiences before diagnosis. Access to other patients' lived experiences with the diagnostic process and with cancer, and an enhanced supportive role of family doctors might improve outcomes for patients and families.

Introduction

Cancer is the leading cause of death in Canada [1]. About half of Canadians will develop cancer in their lifetime, and about one-fourth will die of the disease [2]. Evidence suggests that diagnosing cancer at earlier stages may be associated with improvements in survival [3].

Significant intervals from first noticing a symptom to receiving a cancer diagnosis (known as the diagnostic period) have been widely documented in the literature [4, 5], and related to later stages at diagnosis, as well as suboptimal survival, quality of life post-treatment and patient experience [3, 6]. In Alberta, analyses of administrative data spanning 2004-2011 described potentially preventable long periods from suspicion to diagnosis for breast, colorectal, and lung cancers [7-10]. In Canada and across the world there are substantial variations in the length of the diagnostic period for different cancers [4, 11], and numerous studies have focused on understanding factors that may influence this timeline in order to improve health outcomes and patient experience [12].

The importance of discussing the path to diagnosis from the perspective of patients and family members is increasingly acknowledged in the literature [13]. Receiving a cancer diagnosis is often preceded by a period of waiting for a diagnosis following the discovery of symptoms, which is anxiety-provoking [14]. The psychosocial impacts of the wait may be accentuated if patients believe there have been inefficiencies in their pathway to diagnosis. In Canada, discussions to advance cancer diagnosis are mostly based on timeline-related information available in administrative databases [9, 10], and not so much on perceived timelines and patient and family experiences [6, 15]. This study was designed to contribute to filling that gap. The objective was to examine the perspectives of a group of patients and family members in Alberta, Canada, on factors associated with timelines to diagnosis and overall patient and family experience. Learning more about perceptions and experiences in the diagnostic period may help inform the development of interventions to minimize stress and improve satisfaction with care, which may have a significant impact on wellbeing [16].

Methods

Participants

Participants were associated with the Patient & Family Advisor Network (PFAN) of CancerControl Alberta (Alberta Health Services). PFAN is a community of people who are committed to using their experiences to help improve the health system. Participants in this study were patients with cancer or relatives of patients with cancer who had received a cancer diagnosis within the last three years, and were living in Alberta at the time of diagnosis.

To recruit participants, the PFAN coordinator sent all PFAN members an electronic engagement request inviting expressions of interest in participating in the study. The PFAN coordinator then sent interested members an email with information about the study and a screening questionnaire for them to complete. Responses from the questionnaire were used to purposefully select participants with different types of cancer, within different sex and age ranges, assigned to curative or non-curative treatment, and residing across Alberta (rural and urban) to ensure a variety of cancer experiences [17]. The PFAN coordinator invited selected participants to be part of the study and sent them an email that included a consent form to be reviewed prior to the interview. Additional participants were invited to participate in the study until data saturation was reached [17]. In total, 20 interviews were conducted; with the participation of 18 patients and five family members.

Procedure

The framework used for the study was 'Model of Pathways to Treatment' [18-20]. It identifies four intervals from suspicion of a health problem to receiving treatment: 1) from detection of symptoms to perceiving a reason to discuss symptoms with a healthcare provider (*appraisal*); 2) from perceiving a reason to discuss symptoms with a healthcare provider to first consultation (*help-seeking*); 3) from first

consultation to formal diagnosis, including the initiation of investigation, prescription of tests, examinations, and diagnosis (*diagnosis*); and, 4) from formal diagnosis to start of treatment (*pre-treatment*) [19]. The study covers the first three intervals, referred to collectively as the diagnostic period.

The study used a qualitative research approach [17, 21]. This method was selected to facilitate exploration of perspectives and experiences to capture new insights that may inform how to improve experiences during cancer diagnosis, complementing the previous quantitative approaches undertaken in Alberta. In-depth, semi-structured interviews were used for data collection. Interviews followed an interview guide that was developed in close collaboration with PFAN leadership and with feedback from patient advisors affiliated with the Cancer Strategic Clinical Network (S1 and S2 Appendices). Using accepted qualitative research standards [22], pilot interviews were conducted with four patients with cancer to ensure the interview guide answered the proposed research objective. The interview guide covered topics such as how participants made sense of their symptoms, why they chose to visit a healthcare provider and how they experienced going through appointments, referrals, and tests before they were provided with a definitive cancer diagnosis. It also included a section on recommendations for improvement including the need for emotional support during the diagnostic period.

The study was conducted with written ethics approval granted by the Health Research Ethics Board of Alberta –Cancer Committee (Study ID: HREBA.CC-18-0210). Interviews were conducted by APB, a qualitative researcher by background, with a PhD in social sciences, with interest in the diagnostic period and no previous work in the area with patients or family relatives. She was involved in the early development of the study, and had no prior relationship or sharing of personal information with the participants approached for interview. Interviews were conducted face-to-face at a time and location

convenient for each participant. Most participants were interviewed in a room at their closest cancer centre, and three in a meeting room in the facility where the PFAN coordinator worked. In one case it was not possible to find a convenient location and the interview was conducted through videoconference. There was no presence of non-participants during the interviews. Before proceeding with each interview, participants were invited to sign the consent form that they had received from the PFAN coordinator by email. Interviews took place between June 26 and September 7, 2018, and lasted an average of 41 minutes (range 29-88 minutes). During each interview the researcher took field notes to maintain contextual details and non-verbal expressions. All interviews were audio-recorded and subsequently transcribed verbatim. To protect the identity of participants, at the transcription stage each interview was assigned an identification number and any identifying information was deleted.

Analysis

Interview transcripts were imported into NVivo Version 11 (QSR International, Australia). Transcripts were thematically analyzed using an inductive data-driven coding process to reflect on how participants made meaning of their experiences without predetermined theories [23]. This process entailed a methodological review of the full text of each interview transcript to detect recurrent themes that were then organized into a set of codes that were systematically applied to fragments of text [21]. The researcher who conducted the interviews did all of the coding. To ensure consistency and trustworthiness [23], a second researcher coded randomly-selected segments of text. The two researchers discussed their interpretation and codes until they reached consensus. To increase validity, participants were sent research findings and given the opportunity to provide feedback (by email or phone), and findings and feedback were validated in a subsequent group discussion with eight patients with cancer [23].

Results

Patients with cancer and family members who participated in the study represented ten different cancers, 74% of them were women (n=17), their median age was 59 years (range 42-76), 70% resided in urban locations (n=16), 65% of patients had been treated with curative intent (n=15), and the median time between the date of diagnosis and the date of the interview was 19 months (range 2-36). Participants had diverse experiences during the diagnostic period, but some commonalities exist. Thematic analysis revealed three salient themes as being relevant to their experience (S3 Fig): potentially avoidable delays, concerns about health status, and misunderstood investigation process. Participants struggled emotionally in the period between identifying symptoms and receiving a cancer diagnosis, and had suboptimal care experiences.

S3 Fig. Emergent themes relevant to cancer patients and family members' experience during the diagnostic period

Potentially avoidable delays

Participants referred to potentially avoidable long periods of time spent in the diagnostic period. They mentioned delays related to the patient, to the doctor, and to the health system.

Patient-related delays. Initial inaction by patients caused delays. Patients did not initially act because they did not identify symptoms or did not think symptoms were signs of a serious problem. An additional cause of delay was that patients postponed visits with their doctor due to fear of cancer, being busy, feeling embarrassed or not feeling it was an appropriate use of the doctor's time.

Having some degree of awareness about the seriousness of symptoms, and knowing individuals who had experienced cancer played a very important role in acknowledging the problem and deciding to go to

the doctor promptly after noticing a symptom. As this participant explained it: “A friend from the community had [breast cancer] and died of it. So, I felt this lump. As soon as we got home, I made an appointment” [breast cancer patient 10].

Family doctor-related delays. In some cases, participants reported that they believed that their doctor’s inability to identify symptoms led to patients undergoing investigation for diseases other than cancer, or a later visit to the emergency department. One participant explained this delay in eventually investigating and identifying cancer as: “she just developed this cough. It wouldn’t go away. We took her to the doctor and just said it’s allergies […]. Several months went by, and she wasn’t any better. We went to the doctor again and again before even thinking of cancer” [relative of lung and brain cancer patient 12].

Participants perceived family doctors as the patients’ doorway to the diagnostic pathway. In the context of their experience, some participants referred to their family doctor as the person who restricted access to the investigation of cancer symptoms and jeopardized a timely diagnosis. One explained: “I guess it couldn’t be cancer if [the doctor] didn’t even check for it” [breast cancer patient 2], and:

> “It isn’t until [doctors] are convinced something is wrong, nothing is going to happen […]. It was not until we did the private MRI that we found out [she had cancer]. That triggered the doctor really believing that something was wrong and doing something about her cancer” [relative of lung cancer patient 3].

In some cases, participants debated about the role of family doctors and agreed that doctors could play a bigger role at supporting patients in navigating the healthcare system. One participant, reflecting on

her experience, said: “I’ve had this family doctor for many years […]. I felt that he should have been more involved in getting things moving quicker” [colon cancer patient 14].

System-related delays. Long waiting times to see a specialist, undergo testing or learn test results delayed diagnosis. Some participants spent extensive time following the multiple steps involved in the investigation. They shared frustration with what they reflected to be an uncoordinated and inefficient process. A few shared that there was not much they could have done about it, while others expressed that “there’s a responsibility for [patients] to be more proactive” [ovarian cancer patient 4]. As explained by this participant:

> “In my case, people doing the test didn’t follow through. So, that process was dropped. But I still had those symptoms, so we should have done more investigation. I had to be assertive enough to say to the doctor: ‘I need this exam’” [colon cancer patient 15].

Being familiar with cancer and having system connections or being knowledgeable about how the system works were mentioned as enablers for self-advocacy during the diagnostic period and prompt diagnosis. One participant explained, for example:

> “I was phoning and trying not to harass, but I knew what my options were, what I can do. I remember phoning my family doctor and, you know, ‘can you get us in?’ to get a colonoscopy earlier. Unless you are an advocate, you get lost in the system” [relative of lung cancer patient 2].

Concerns about health status

Participants mentioned that it was stressful to consider the possibility of a major health condition such as cancer. In most cases, family members were mentioned as the primary source of emotional support. Some participants also assigned an emotional supporting role to family physicians: “they could be the

hub, supporting the patient" [prostate cancer patient 5]. In terms of the timing of health concerns, most participants mentioned that before the investigation "you want to trivialize it, it's not a big deal" [breast cancer patient 1], but once the investigation process started, they began to consider the possibility of a serious health problem.

Participants shared three elements that triggered being concerned about a serious health problem. First, the fact that their family doctor decided to pursue testing or referral to a specialist prompted worries. "I thought: 'nobody was too worried about it until now, why do I have to go [to the specialist] suddenly?' So I became worried" [anal cancer patient 18]. Second, the realization that the investigation was not as straightforward as they thought caused concerns. "It was scary; Dr. [name] sent us for blood tests again, and the scans, that was like two days" [relative of prostate cancer patient 5]. Third, the existence or non-existence of communication with healthcare providers during the investigation caused patients to worry about their health. "I was having the mammogram, the radiologist was there. She showed me what she saw on the screen and she said: 'this is what I am looking at', and I'm concerned about that" [breast cancer patient 17]. "A lot of cancer people say that at the radiology place, when they are doing [a mammogram], the radiologist often comes in and explains; they can often tell right away. But nothing, that did not happen, and I'm worried" [breast cancer patient 1].

Misunderstood investigation process

The process of investigation was described differently by participants who accessed care through the emergency department and those who accessed care through their family doctor. Participants who went to the emergency department described the investigation as "very abrupt, instantaneous and with no stress, because it was right away" [breast cancer patient 2]. In several cases, participants who saw their doctor and had the investigation initiated by their doctor explained that the investigation was cumbersome and stressful. They referred to specific complications such as having tests done multiple

times or having to travel to inconvenient locations for medical appointments, and also to the fact that it involved multiple steps that were often unexpected and seemed uncoordinated. As explained by this participant: “I just thought I was gonna go in for a mammogram […]. I went for my mammogram and I thought everything would be fine, but they called me back and they said: ‘we need to see you again’” [breast cancer patient 10]. What seemed particularly stressful for participants was the fact they did not understand what the investigation process entailed, and were unsure about what to expect. As explained by one participant:

> “There didn’t seem to be a plan, really. I mean, I know they have a plan because that’s what they’ve done a million times before, but they don’t share that too much other than to say: ‘we are going to do a biopsy or whatever test’. There is not a whole lot of explanation […]. I was just following what they were saying, and I was: ‘ok, I’ll just show up there for that appointment and do that’” [breast cancer patient 16].

After reflecting on how challenging it had been for them to navigate the investigation process, some participants stressed that others might struggle. In particular, they referred to patients from cultural minorities, elderly, with less education or other characteristics that may impact their ability to advocate for themselves. “Where am I supposed to go? People don’t have a clue; […] you feel lost, it’s very scary”, and “I kept saying: ‘if you were an immigrant coming in here, how would you ever figure all this out?’” [anal cancer patient 18]. Participants emphasized the importance of having family members who can support patients, and the need to improve the coordination of services and have resources available to patients: “whether it be a nurse navigator, whether it be some written information, or like just somebody to tell you: ‘this is what [the investigation process] looks like and these are the timelines’” [breast cancer patient 6].

Discussion

The study described the diagnostic period from the perspective of a group of patients with cancer and family members in Alberta. Findings contribute to the literature by focusing on perceived challenges along the pathway to diagnosis, and thus may have implications for the development of interventions to improve the experiences of patients and family members. Of relevance, findings showed that patients and family members participating in the study experienced anxiety and had suboptimal experiences. Participants expressed the importance of self-advocacy and wanting to have an active role in their care, and suggested unmet supportive care needs when navigating the system. These findings highlight a need for further discussion on the provision of psychosocial supports to potential cancer patients and their families before they have a confirmed diagnosis of cancer.

The finding of participants feeling distressed during this period is consistent with previous studies undertaken in various countries including the United Sates, Denmark and Canada [13, 18]. Before diagnosis, individuals with symptoms suspicious of cancer face uncertainty and threat of a serious illness while having to undergo medical appointments and tests, which might be fearsome, uncomfortable and demanding. Findings from the study are also in alignment with the literature by indicating that fear about having a serious health condition is an important cause of anxiety among patients and family members waiting for diagnosis [24]. Also in accordance with the literature, findings showed that perceived long timelines add stress to an already stressful situation [25]. Consistent with previous research, participants associated delays with their inaction or late action in seeking medical attention [6], with their doctor's failure to correctly identify cancer symptoms [12, 26], and with system inefficiencies including variable access to specialists and testing and limited coordination of care [27, 28]. Of relevance, participants in the study referred to cancer awareness and second-hand experience

with cancer as two important elements supporting the patient's prompt consult with a doctor. While the relevance of cancer awareness has been previously acknowledged [29, 30], the influence of shared cancer stories on the experiences of potential cancer patients with the diagnostic period has not been discussed in the literature. In the post-diagnosis literature, access to patients' real experiences with the disease has been reported important in providing general support to patients with cancer including the provision of information related to cancer and care process, and emotional help [31, 32]. The potential role of the socialization of these experiences in not just helping individuals seek medical attention, but also dealing with information and emotional aspects of a prospective cancer diagnosis at the beginning of their journey requires further investigation.

A relevant contribution from our study is the importance of self-advocacy and the need to better support patients and family members acquire an active role in their care prior to receiving a cancer diagnosis. Similar to what has been documented in the post-diagnosis literature [33], participants in the study referred to the concept of 'patient activation' [34]. They articulated their need or willingness and ability to take independent actions to manage their health and care throughout the diagnostic period. Similar to what has been discussed in the context of treatment and survivorship [35], the healthcare system, even in the pre-diagnosis period, is increasingly complex, with a growing number and variety of specialties, care delivery sites, and diagnostic tests. In the face of navigating these complexities, and in the context of the psychological burdens of potentially facing cancer it is not surprising that many patients and families find crucial to advocate for getting their care needs met [32]. In order to fully benefit from the care available to them and have improved experiences, patients and families promote their own interests and actively try to avoid situations such as delayed appointments with a specialist or delayed access to appropriate testing.

As acknowledged by participants and supported by previous research, in order to be able to advocate for themselves, patients and family members need to be involved with individuals and groups that support their interests [32, 33]. Unlike post-diagnosis, before diagnosis patients are not “cancer patients” yet and they and their family members do not hold membership into particular patient/family groups that can provide this support, leaving healthcare providers as the main group who can do it. While at post-diagnosis stages oncology care teams are an important source of support to patients and families [36], before diagnosis patients expect this role to be played by family doctors. Family doctors in Canada play a key role in helping to manage and coordinate care for patients before and after diagnosis [37], and as suggested by our findings their engagement from the beginning of the patients’ journey in helping them navigate the diagnostic period and advocating for them when required is important. We heard from participants that it is important for patients/families to feel that family doctors are on their side and support them. Family doctors can support patient and family members willing to engage in self-advocacy behaviours by trying to establish deeper connections with them and adequately informing them. As reported in previous research, family doctors are a primary source of information during the diagnostic period [15], and patients need to feel adequately informed in order for them to participate effectively in making decisions throughout the diagnostic period [32]. Based on our findings, optimal attention to patient and family needs may include hearing their concerns, providing them with regular updates on the investigation process, engaging in discussions of expectations about the different steps involved in the diagnostic period, and assessing their understanding of these conversations. Finding ways to promote and support family doctors in the care of potential cancer patients throughout the diagnostic period and equipping them with the skills and tools required to better support them should be explored further.

Limitations

The findings of this study should be considered in light of some limitations. First, the study was not longitudinal and results are particular to the time frame the interviews were conducted. Distress and recovery from the diagnostic period is a dynamic process, the perspectives of patients and family members would likely be different at an earlier or later point in time. Their perceptions may have changed as more time elapsed since the date of diagnosis. Second, the information was collected retrospectively and data might be subject to memory recall errors. Finally, as in any study involving self-reported data, results may be subject to exaggeration and attribution. Despite these limitations, results from this exploratory study provide important contributions to this area given the limited number of studies focused on perceived timelines and challenges during the diagnostic period.

Conclusion

In an effort to streamline the diagnostic period in Alberta, Alberta Health Services and other stakeholders have established streamlined diagnostic programs for breast, lung and prostate cancers [38-40]. The Cancer Strategic Clinical Network is currently initiating a program to establish an Alberta Facilitated Cancer Diagnosis Strategy that goes across cancer sites and geographies in the province. Our study asked patients and family members in the province about their experience from symptom to cancer diagnosis and found that they struggled emotionally. Findings complement quantitative studies that described long and variable periods from suspicion to diagnosis for different cancers [8-10]. Findings suggested that from the perspectives of patients and family members shorter times to diagnosis are desirable, but many additional factors also need to be considered for improved satisfaction with care. Supporting individuals deal with a prospective cancer diagnosis by facilitating access to lived experiences with the diagnostic process and cancer, and enhancing the supportive role of

family doctors may improve outcomes for patients and family members. Although this study helps shed light on how to improve the experiences of patients and family members during the diagnostic period, more work is required to understand how some of the psychosocial supports available to cancer patients after diagnosis could be used to support these patients and their families before diagnosis.

Acknowledgements

The authors acknowledge the Patient & Family Advisor Network (CancerControl Alberta) for their assistance in recruiting participants, and feedback on data collection protocol; Debora Allatt, Lorelee Marin, Frances Cusano, and Charlie Fischer for their feedback; and study participants for their insights.

References

1. Statistics Canada. The 10 leading causes of death, 2012. Ottawa, ON: Statistics Canada; 2015 [cited 2018 January 9]; Available from: http://www.statcan.gc.ca/pub/82-625-x/2015001/article/14296-eng.htm.

2. Canadian Cancer Statistics Advisory Committee. Canadian cancer statistics 2019. Toronto, ON: Canadian Cancer Society2019.

3. Neal RD, Tharmanathan P, France B, Din NU, Cotton S, Fallon-Ferguson J, et al. Is increased time to diagnosis and treatment in symptomatic cancer associated with poorer outcomes? Systematic review. Br J Cancer2015 Mar 31;112:S92-S107.

4. Butler J, Foot C, Bomb M, Hiom S, Coleman M, Bryant H, et al. The International Cancer Benchmarking Partnership: An international collaboration to inform cancer policy in Australia, Canada, Denmark, Norway, Sweden and the United Kingdom. Health Policy2013 Sep;112(1-2):148-55.

5. Maringe C, Walters S, Rachet B, Butler J, Fields T, Finan P, et al. Stage at diagnosis and colorectal cancer survival in six high-income countries: A population-based study of patients diagnosed during 2000-2007. Acta Oncol2013 Jun;52(5):919-32.

6. Brousselle A, Breton M, Benhadj L, Tremblay D, Provost S, Roberge D, et al. Explaining time elapsed prior to cancer diagnosis: Patients' perspectives. BMC Health Serv Res. [Article]. 2017 Jun;17:448.

7. Li X, Scarfe A, King K, Fenton D, Butts C, Winget M. Timeliness of cancer care from diagnosis to treatment: A comparison between patients with breast, colon, rectal or lung cancer. International Journal of Quality Health Care2013 Apr;25(2):197-204.

8. Kim JO, Davis F, Butts C, Winget M. Waiting time intervals for non-small cell lung cancer diagnosis and treatment in Alberta: Quantification of intervals and identification of risk factors associated with delays. Clin Oncol2016 Dec;28(12):750-9.

9. Yuan Y, Li M, Yang J, Elliot T, Dabbs K, Dickinson JA, et al. Factors related to breast cancer detection mode and time to diagnosis in Alberta, Canada: A population-based retrospective cohort study. BMC Health Serv Res2016 Feb 19;16.

10. Sikdar KC, Dickinson J, Winget M. Factors associated with mode of colorectal cancer detection and time to diagnosis: A population level study. BMC Health Serv Res2017 Jan 5;17.

11. Grunfeld E. It takes a team CanIMPACT: Canadian team to improve community-based cancer care along the continuum. Can Fam Physician. [Editorial Material]. 2016 Oct;62(10):781-2.

12. Weller D, Vedsted P, Anandan C, Zalounina A, Fourkala EO, Desai R, et al. An investigation of routes to cancer diagnosis in 10 international jurisdictions, as part of the International Cancer Benchmarking Partnership: Survey development and implementation. BMJ Open2016 2016;6(7).

13. Miles A. The psychological implications of diagnostic delay in colorectal cancer patients. In: Olsson L, editor. Timely Diagnosis of Colorectal Cancer Cham, Switzerland: Springer International Publishing; 2017. p. 103-19.

14. Brocken P, Heijden EHFM, Oud KTM, Bootsma G, Groen HJM, Donders ART, et al. Distress in suspected lung cancer patients following rapid and standard diagnostic programs: A prospective observational study. Psychooncology. [Article]. 2015;24(4):433-41.

15. Wiljer D, Walton T, Gilbert J, Boucher A, Ellis PM, Schiff S, et al. Understanding the needs of lung cancer patients during the pre-diagnosis phase. J Cancer Educ2012;27(3):494-500.

16. Jonikas J, Grey D, Copeland M, Razzano L, Hamilton M, Floyd C, et al. Improving propensity for patient self-ddvocacy through wellness recovery action planning: Results of a randomized controlled trial. Community Ment Health J2013;49(3):260-9.

17. Patton MQ. Qualitative research & evaluation methods. Integrating theory and practice. Saint Paul, MN: SAGE Publications, Inc; 2015.

18. Weller D, Vedsted P, Rubin G, Walter FM, Emery J, Scott S, et al. The Aarhus statement: Improving design and reporting of studies on early cancer diagnosis. Br J Cancer2012 Mar 27;106(7):1262-7.

19. Walter F, Webster A, Scott S, Emery J. The Andersen Model of Total Patient Delay: a systematic review of its application in cancer diagnosis. J Health Serv Res Policy2012 Apr;17(2):110-8.

20. Scott SE, Walter FM, Webster A, Sutton S, Emery J. The model of pathways to treatment: conceptualization and integration with existing theory. British Journal of Health Psychology2013 Feb;18(1):45-65.

21. Denzin N, Lincoln Y. The SAGE handbook of qualitative research 5th ed. Thousand Oaks, CA: SAGE Publications, Inc; 2018.

22. Creswell J. Research design: Qualitative, quantitative, and mixed methods approaches: Thousand Oaks: Sage Publications; 2014.

23. Miles MB, Huberman MA, Saldana J. Qualitative data analysis: A methods sourcebook. 3rd ed. Thousand Oaks, CA: SAGE Publications, Inc; 2014.

24. Brocken P, Prins JB, Dekhuijzen PNR, van der Heijden HFM. The faster the better?-A systematic review on distress in the diagnostic phase of suspected cancer, and the influence of rapid diagnostic pathways. Psychooncology. [Article]. 2012;21(1):1-10.

25. Miles A, McClements PL, Steele RJC, Redeker C, Sevdalis N, Wardle J. Perceived diagnostic delay and cancer-related distress: A cross-sectional study of patients with colorectal cancer. Psychooncology. [journal article]. 2017;26(1):29-36.

26. Macleod U, Mitchell ED, Burgess C, Macdonald S, Ramirez AJ. Risk factors for delayed presentation and referral of symptomatic cancer: Evidence for common cancers. Br J Cancer2009 Dec 3;101 Suppl 2:S92-s101.

27. Barisic A, Kish M, Gilbert J, Mittmann N, Moineddin R, Sisler J, et al. Family physician access to and wait times for cancer diagnostic investigations: Regional differences among 3 provinces. Can Fam Physician. [Article]. 2016 Oct;62(10):E599-E607.

28. Brouwers MC, Vukmirovic M, Tomasone JR, Grunfeld E, Urquhart R, O'Brien MA, et al. Documenting coordination of cancer care between primary care providers and oncology specialists in Canada. Can Fam Physician. [Article]. 2016 Oct;62(10):E616-E25.

29. Richards MA. The national awareness and early diagnosis initiative in England: Assembling the evidence. Br J Cancer2009 Dec;101:S1-S4.

30. Quaife SL, Forbes LJL, Ramirez AJ, Brain KE, Donnelly C, Simon AE, et al. Recognition of cancer warning signs and anticipated delay in help-seeking in a population sample of adults in the UK. Br J Cancer. [Article]. 2014(1):12.

31. Lawler M, Prue G, Banks I, Law K, Selby P, McVie G, et al. Mapping the cancer patient information landscape: A comparative analysis of patient groups across Europe and North America. Eur J Cancer. [Article]. 2018 03/01/March 2018;92:88-95.

32. Hagan TL, Rosenzweig MQ, Zorn KK, van Londen GJ, Donovan HS. Perspectives on self-advocacy: Comparing perceived uses, benefits, and drawbacks among survivors and providers. Oncol Nurs Forum2017;44(1):52-9.

33. Hagan TL, Donovan HS. Self-advocacy and cancer: A concept analysis. J Adv Nurs2013;69(10):2348-59.

34. Hibbard JH, Stockard J, Mahoney ER, Tusler M. Development of the Patient Activation Measure (PAM): Conceptualizing and Measuring Activation in Patients and Consumers. Health Serv Res. [Article]. 2004;39(4p1):1005-26.

35. Gruman J, Rovner MH, French ME, Jeffress D, Sofaer S, Shaller D, et al. From patient education to patient engagement: Implications for the field of patient education. Patient Educ Couns. [Article]. 2010 1/1/2010;78:350-6.

36. Brown R, Butow P, Wilson-Genderson M, Bernhard J, Ribi K, Juraskova I. Meeting the decision-making preferences of patients with breast cancer in oncology consultations: Impact on decision-related outcomes. J Clin Oncol2012;30(8):857-62.

37. Easley J, Miedema B, O'Brien MA, Carroll J, Manca D, Webster F, et al. The role of family physicians in cancer care: Perspectives of primary and specialty care providers. Current Oncology. [Article]. 2017 Apr;24(2):75-80.

38. Prostate Cancer Centre Calgary. Rapid Access Clinics (RAC). 2016 [cited 2019 September 9]; Available from: http://www.prostatecancercentre.ca/our-programs/patient-services/#rapid-access-clinics.

39. Tremblay A, Strilchuk N, Taghizadeh N, Fortin M, Burrowes P, Hampton L, et al. Radiologist initiated specialty referral for patients suspected of having a thoracic malignancy. Canadian Journal of Respiratory, Critical Care, and Sleep Medicine2017;1(4):180-5.

40. Alberta Health Services. Comprehensive Breast Care Program. Alberta Health Services; 2018 [cited 2018 January 22]; Available from: https://www.albertahealthservices.ca/info/service.aspx?id=1022658.

Supporting information

S1 Appendix. Semi-structured interview protocol (patients)

S2 Appendix. Semi-structured interview protocol (family members)

S3 Fig. Emergent themes relevant to cancer patients and family members' experience during the diagnostic period

 Click here to download Figure S3_Fig.tiff

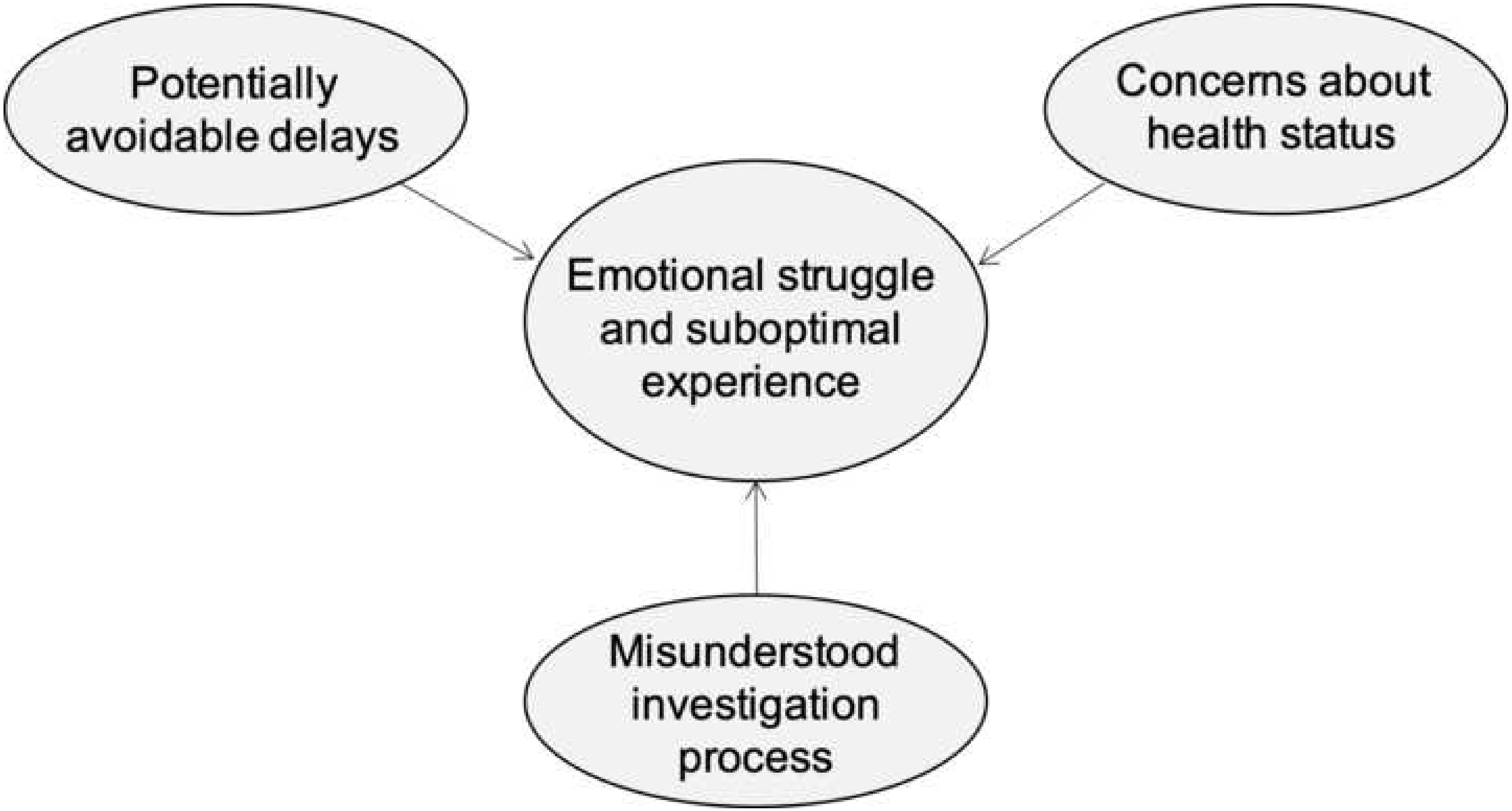

Supporting infromation (file 1)

Click here to access/download
Supporting Information
S1_Appendix.docx

Supporting infromation (file 2)

Click here to access/download
Supporting Information
S2_Appendix.docx
