# Supplementary material for: From symptom to cancer diagnosis: Perspectives of patients and family members in Alberta, Canada
Source: PLoS One. 2020 Sep 24;15(9):e0239374. doi: 10.1371/journal.pone.0239374 (PMC7514000; doi:10.1371/journal.pone.0239374)
Supplement: Attachment — Submitted filename: Response to Reviewers.docx [file pone.0239374.s007.docx]

**Manuscript** PONE-D-20-12776

**Response to Reviewers**

Dear Dr. Galli,

Thank you for giving us the opportunity to submit a revised draft of the manuscript “From symptom to cancer diagnosis: Perspectives of patients and family members in Alberta, Canada” for publication in PLOS ONE. We appreciate the time and effort that you and the reviewers dedicated to providing feedback on our manuscript and are grateful for the insightful comments on and valuable improvements to our paper. We have incorporated all the suggestions made by the Reviewer #3. Those changes are highlighted within the manuscript. Please see below, in blue, for a point-by-point response to their comments. All line numbers refer to the revised manuscript file with tracked changes.

**Reviewer #3**

**Comment 1**. I did not review this manuscript in the first round. However, it appears that the authors have been responsive to all of the comments of the original reviewers.

**Authors’ response**. Thank you.

**Comment 2**. I only have one technical comment. In the description of trustworthiness (Methods), the authors say that a second investigator coded a random sample of segments and that disagreements were settled via consensus. However, this tells us little about how often two skilled coders would come to similar conclusions. Either present information on reliability or list this as a limitation.

**Authors’ response**. As suggested by the reviewer, we have added information on reliability: *An interrater reliability analysis using Cohen’s Kappa was performed for 20% of the coded interviews. The Kappa value was 0.81, which can be interpreted as “strong agreement”* [1] (line numbers 170-172).

**Comment 3**. Also, at the bottom of page 13, the sentence that starts "the role of socializing patients' experiences with cancer..." does not make sense to me. What does it mean to "socialize" experiences? Please clarify or cut.

**Authors’ response**. Thank you for pointing this out. We have clarified what we meant by ‘socialize’ by editing the text at the bottom of page 13. The sentence now starts “the role of accessing patients’ experiences with cancer…” (line number 317). The word ‘socialize’ was also used in another sentence that we have also edited (line numbers 341-343):

*The potential role of having access to these experiences in not just helping individuals seek medical attention, but also dealing with information and emotional aspects of a prospective cancer diagnosis at the beginning of their journey requires further investigation.*

**References:**

1.	McHugh ML. Interrater reliability: The kappa statistic. Biochemia Medica. 2012;22(3):276-82. PubMed PMID: 82559661.
